# Spatial Distribution of Glucan Type and Content between Caps and Stalks in *Pleurotus eryngii*: Impact on the Anti-inflammatory Functionality

**DOI:** 10.3390/ijms19113371

**Published:** 2018-10-28

**Authors:** Vaclav Vetvicka, Ofer Gover, Hilla Hayby, Ofer Danay, Nirit Ezov, Yitzhak Hadar, Betty Schwartz

**Affiliations:** 1 University of Louisville, Department of Pathology, University of Louisville, Louisville, KY 40202, USA; v0vetv01@louisville.edu; 2 Institute of Biochemistry, School of Nutritional Sciences, Food Science and Nutrition, The Robert H. Smith Faculty of Agriculture, Food and Environment, The Hebrew University of Jerusalem, Rehovot 76100, Israel; ofer.gover@mail.huji.ac.il (O.G.); hilla.hayby@mail.huji.ac.il (H.H.); 3 Edible Mushrooms, MIGAL, 11016 Kiryat Shmona, and Tel Hai College, 12210 Upper Galilee, Israel; ofer@migal.org.il; 4 Edible Mushrooms Development, MIGAL, Kiryat Shmona 11016, Israel; niritezov@walla.co.il; 5 Department of Plant Pathology and Microbiology, The Robert H. Smith Faculty of Agriculture, Food and Environment, The Hebrew University of Jerusalem, Rehovot 76100, Israel; yitzhak.hadar@mail.huji.ac.il

**Keywords:** *P. eryngii*, glucans, inflammation, inflammatory bowel disease

## Abstract

*Pleurotus eryngii* is recognized for its prominent nutritional and medicinal value. In our study, we tested the effect of glucans on lipopolysaccharide (LPS)-induced production of TNF-α. We demonstrated that glucan extracts are more effective than mill mushroom preparations. Additionally, the effectiveness of stalk-derived glucans were slightly more pronounced than of caps. Cap and stalk glucans from mill or isolated glucan competed dose-dependently with anti-Dectin-and anti-CR-3 antibodies, indicating that they contain β-glucans recognized by these receptors. Using the dextran sulfate sodium (DSS)-inflammatory bowel disease mice model, intestinal inflammatory response to the mill preparations was measured and compared to extracted glucan fractions from caps and stalks. We found that mill and glucan extracts were very effective in downregulating IFN-γ and MIP-2 levels and that stalk-derived preparations were more effective than from caps. The tested glucans were equally effective in regulating the number of CD14/CD16 monocytes and upregulating the levels of fecal-released IgA to almost normal levels. In conclusion, the most effective glucans in ameliorating some IBD-inflammatory associated symptoms induced by DSS treatment in mice were glucan extracts prepared from the stalk of *P. eryngii*. These spatial distinctions may be helpful in selecting more effective specific anti-inflammatory mushrooms-derived glucans.

## 1. Introduction

*Pleurotus eryngii* is generally recognized as the king oyster mushroom. It was originally consumed in Europe but is now widely distributed and consumed in other parts of the world including America, Asia, and Africa [1]. The advantageous nutritional and medicinal properties exerted by *P. eryngii* mushrooms are mediated, at least in part, by potent bioactive constituents such as the polysaccharides fraction (glucans) and other molecules that can confer a variety of health benefits such as strong immunomodulatory effects [2,3,4].

The notable regulatory effects of glucans present in the fruiting bodies of edible mushrooms on the immune system have received significant attention and extensive studies [5,6,7]. The regulating activity of the immune system by mushroom glucans appears to be mediated, at least in part, via direct activation of macrophages and dendritic cells, which are essential components of the innate immune system in mammalians [8].

For glucans to induce their biological response, they should bind to their primary targets; i.e., the inflammatory cells. Several studies have established that, at least for β-glucans, biological effects are exerted through specific binding to the lectin-binding site of complement receptor type three (CR3 (CD11b/CD18)) on immune effector cells and NK cells [8]. β-Glucans may also interact with an additional glucan receptor on inflammatory cells, the Dectin-1 receptor expressed in neutrophils, macrophages, dendritic cells, and some T-cells. Following binding of glucans to Dectin-1 or to CR3 (CD11b/CD18) receptor, several specific activities, such as modulation of phagocytosis, variations of release of inflammatory cytokines, and other activities, are modulated [8].

Inflammatory bowel diseases (IBD) are inflammatory disorders of the intestinal tract and bothersome conditions of unknown etiology that are most common in developed countries, distressing the quality of life of the affected population. There are two types of IBD, Crohn’s disease and ulcerative colitis, which both feature an overactive immune response. Treatment of IBD symptoms is concentrated on the use of nonspecific immunosuppressive therapies (such as steroids), antibiotics, and some novel biologicals therapies mainly targeting the proinflammatory tumor necrosis factor (TNF) pathway [9]. Altogether, these treatments are not always effective in all patients. We believe that ingestion of purified glucans may exert anti-inflammatory effects similar to those previously reported for *P. pulmonarious* glucans [6] and none of the adverse effects of the aforementioned therapies.

We recently reported that *P. eryngii* has the highest total, β- and α-glucan content compared to other Pleurotus species [10]. We also found that the stalks (stipe) of the fruiting body contained higher glucan content than the caps (pileus) [10]. In the present study, we addressed the question whether this spatial distribution has implications on the extent of the anti-inflammatory effect exerted by the two different parts of *P. eryngii* mushroom. We, therefore, concentrated our efforts on investigating whether glucans extracted from stalks and caps are comparable in their anti-inflammatory effects using a wide variety of experimental designs exemplifying inflammatory disorders such as IBD.

## 2. Results

### 2.1. Glucan Content Mushroom Stalk and Cap

The glucan content of the *P. eryngii* was assessed using the Megazyme kit. Total α-glucan and β-glucan content was significantly higher in the stalks of the mushrooms compared to the caps (*p* < 0.001); however, α-glucan content in stalks demonstrated the most significant difference (*p* < 0.0001) (Table 1).

### 2.2. P. eryngii-Derived Glucans Suppressed TNF-α Secretion from Lipopolysaccharide-Treated J774.2 Cells

**Figure 1:**
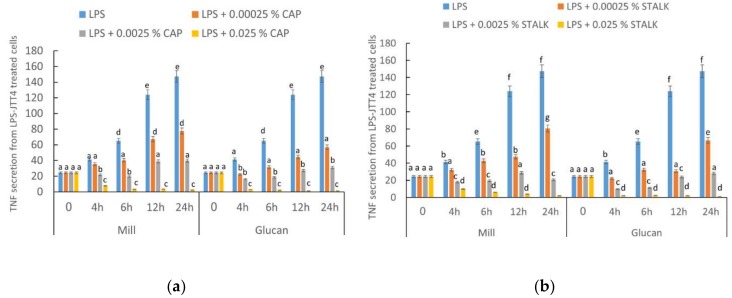
Secretion of lipopolysaccharide (LPS)-stimulated TNF-α by J774.1 (1 × 10^6^ cells/well) macrophages in response to several doses of glucans from mill and isolated glucans from (**a**) caps and (**b**) stalks of *P. eryngii* mushrooms (0.00025%; 0.0025%; 0.025%; i.e., 0.25 or 2.5 or 25 µg/1 mL media). Concentration of TNF-α in the culture supernatants was measured by ELISA and expressed as (pg/mL). Data are expressed as means ± SD. Different letters indicate significantly different values at *p* < 0.05.

TNF-α is an important inflammatory mediator produced from macrophages corresponding to inflammation. Therefore, we used TNF-α as an indicator of macrophage response to lipopolysaccharide (LPS) and glucans. J774.2 cells were incubated with LPS (100 ng/mL) and with/without glucan for 24 h. Supernatant was collected at different intervals (4, 6, 12, and 24 h) and evaluated for TNF-α secretion. Cells were incubated with whole mill or glucan extracts at increasing concentrations. LPS induced TNF-α production in a time-dependent fashion (Figure 1). A significant decrease (∼50%) in TNF-α secretion was observed when 0.00025% extract from cap was added after 4 h and at a concentration of 0.025% TNF-α was hardly detected. TNF-α production was suppressed by both glucans in a dose-dependent manner, showing the greatest consistent effect when glucan extracted from stalks was added. Glucans from glucan extracts exerted more notorious effects than glucans from mill preparations.

### 2.3. Inhibition of Staining of CR3 and Dectin-1 Receptors by P. eryngii Glucans

**Figure 2:**
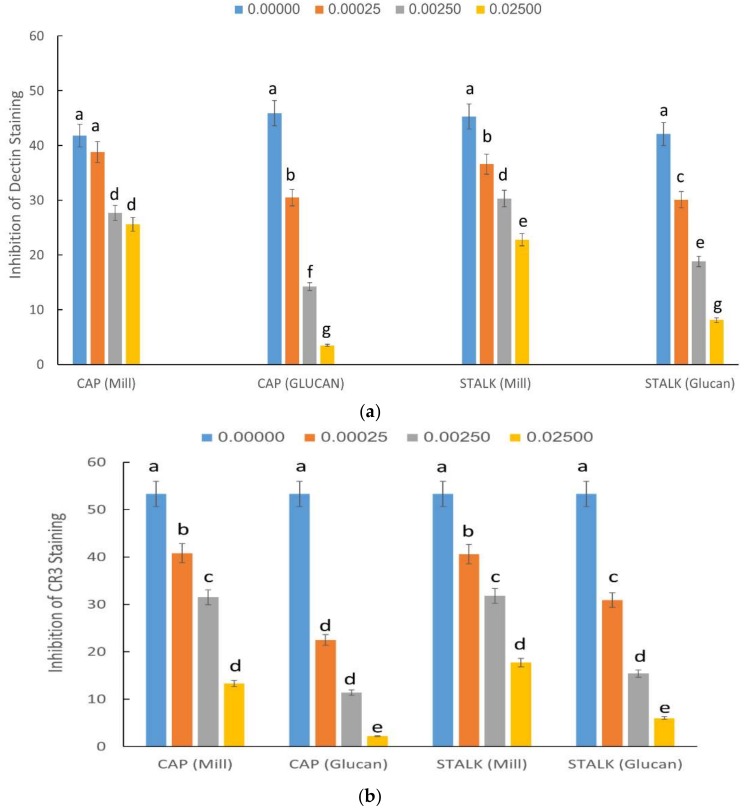
Effect of different concentrations of glucans from mill and isolated glucans from caps and stalks of *P. eryngii* mushrooms (0.00025%; 0.0025%; 0.025%; i.e., 0.25 or 2.5 or 25 µg/1 mL media) on Dectin-1 expressed in human neutrophils (**a**) or CR3 staining expressed in RAW 264.7 cells (**b**). The data shown are the average with error bars indicating the standard deviation. The level of inhibition with increasing concentrations of *P. eryngii* isolated glucans is more potent than that obtained with increasing concentrations of *P. eryngii* mill preparations. No difference between caps and stalks. Different letters indicate significantly different values at *p* < 0.05.

Glucans express their immunostimulating activities via interaction with various surface receptors. The most important of these receptors are Dectin-1 and CR3 (CD18/CD11b). In order to establish the affinity of glucans from different parts of the mushroom to these receptors, the next series of experiments focused on binding to CR3 and Dectin-1 receptors. Our results, summarized in Figure 2, demonstrated that glucan extracts had a strong dose-dependent inhibition on both receptors. Glucan extracts competed with both receptors more efficiently than mill. Glucans extracted from the cap had the strongest inhibition profile on Dectin-1 staining (Figure 2a), but for CR3 binding, glucans extracted from caps and stalks had similar inhibitory activity (Figure 2b).

### 2.4. Effect of Isolated Glucans from Stalk and Cap on Acute Colitis Induction in Mice

**Figure 3:**
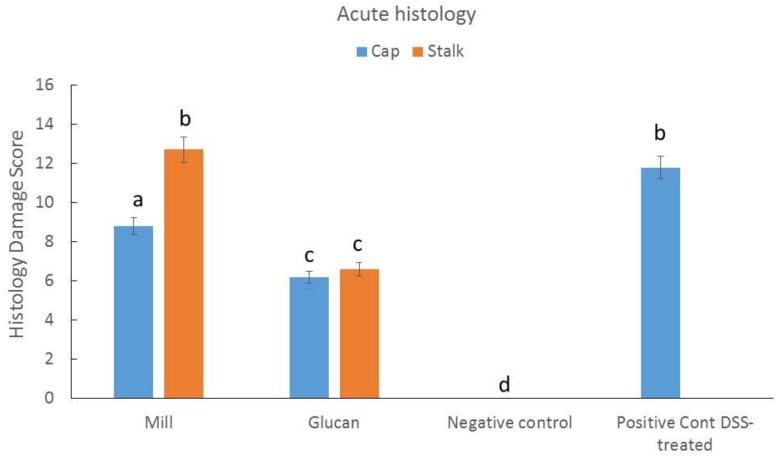
Effect of mill and isolated glucans from caps and stalks of *P. eryngii* mushrooms (1 mg glucan/kg mice BW) on histologic damage score in dextran sulfate sodium (DSS)-induced colitis. DSS was administrated for 7 days and mill and glucan extract treatment started with DSS treatment and continued until day 16 when all mice were sacrificed and tissue samples were taken for analysis. Data represent mean ± SD of six mice per group. Different letters indicate significantly different values at *p* < 0.01.

Histologic damage is one of the most noticeable results from acute DSS-induced colitis in mice. Figure 3 clearly demonstrates that dextran sulfate sodium (DSS) administration resulted in a high histology score of ~12 that was significantly reduced by administration of glucan extracts. Milled mushrooms had lower effects than glucan extracts, and mill prepared from stalk demonstrated almost complete ineffectiveness.

### 2.5. CXCL1, MIP-2, and INF-γ mRNA Expression in Large Intestine

**Figure 4:**
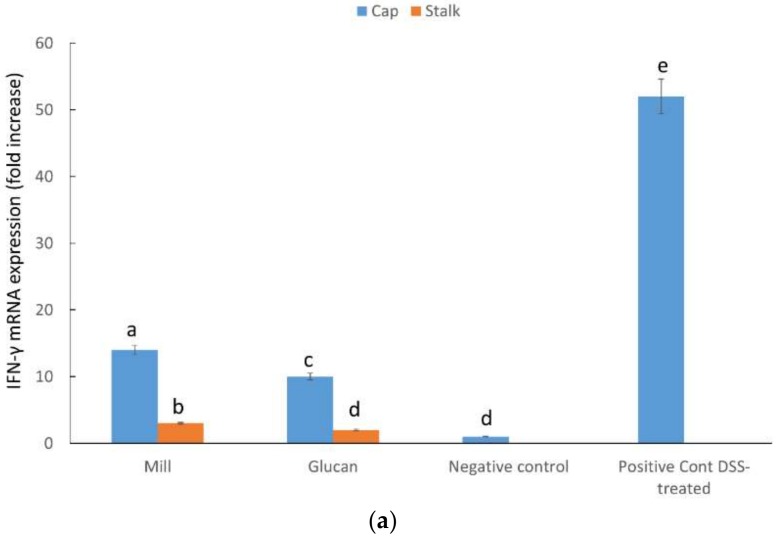

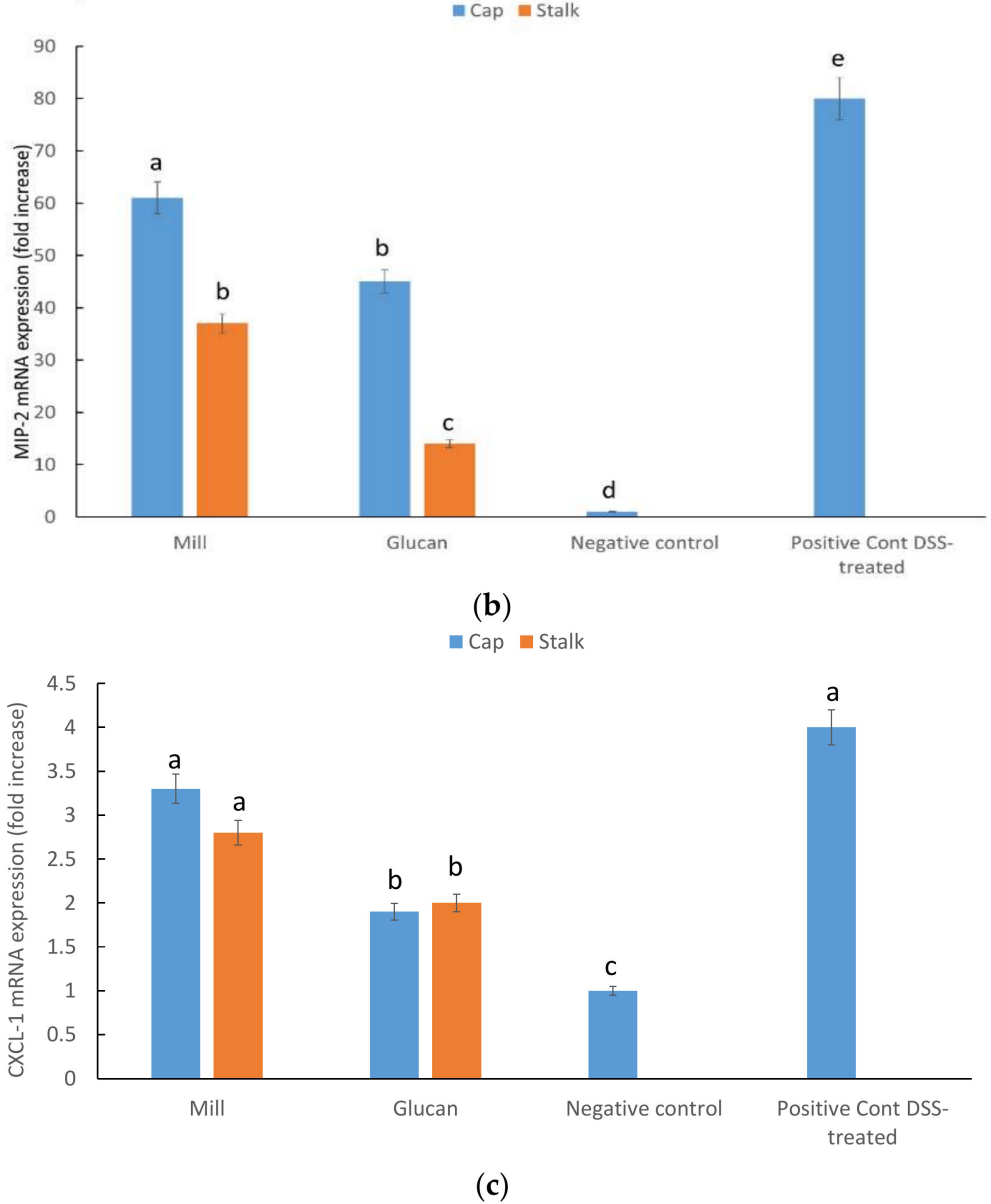
Effect of mill and isolated glucans from caps and stalks of *P. eryngii* mushrooms (1 mg glucan/kg mice BW) on (**a**) INF-γ mRNA levels (**b**) Mip-2 levels mRNA levels and (**c**) CXCL1 mRNA levels in colonic samples relative to negative control (no treatment). DSS was administrated for 7 days and mill and glucan extract treatment started with DSS treatment and continued until day 16 when all mice were sacrificed and tissue samples were taken for analysis. Data represent mean ± SD of six mice per group. Different letters indicate significantly different values at *p* < 0.05.

Expression levels of genes related to immune response were analyzed in samples of large intestine from mice with experimentally-induced acute colitis. INF-γ showed a strong reduction in gene expression by both whole mill and glucan extracts (Figure 4a); although, there was also a significant difference between cap- and stalk-derived glucans. MIP-2 also showed a decrease in expression in treated mice. Mill preparations were less effective than glucan extracts. Additionally, there was also a difference between cap- and stalk-derived glucans with the latter showing stronger effect (Figure 4b). The chemokine CXCL1 showed a significant decrease in expression compared to positive control when mice were treated with glucan extracts but not with whole mill (Figure 4c).

### 2.6. Percentages of CD14/CD16 Monocytes in Colitis

**Figure 5:**
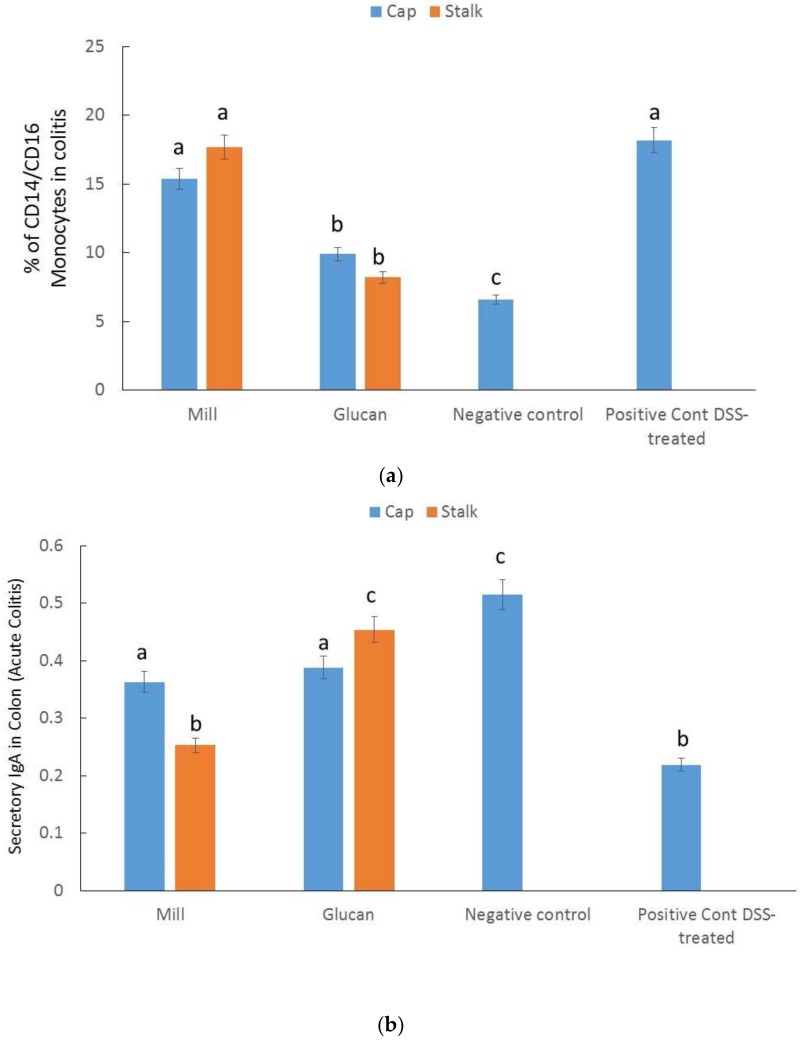
Effect of mill and isolated glucans from caps and stalks of *P. eryngii* mushrooms (1 mg glucan/kg mice BW) on (**a**) percent of CD14^+^/CD16^+^ monocytes and (**b**) on secretory IgA in feces harvested from mice who underwent DSS-induced colitis. Data represent mean ± SD of six mice per group; data were compared between DSS and mill and isolated glucans from caps and stalks of *P. eryngii* mushrooms treated groups. Different letters indicate significantly different values at *p* < 0.05.

Next, we evaluated the percentages of activated monocytes in circulating blood in the different groups (Figure 5a). The maximum percentage (18.2 ± 3%) of activated monocytes was observed in the DSS-induced mice, whole mill had no significant effects, while glucan extracts significantly reduced the percentage of activated monocytes. No difference was detected between caps and stalks.

### 2.7. Secretory IgA in the Colon

Secretory IgA is the most important immunoglobulin secreted towards the mucosal surface in most mammals and play a key role in the protection of the intestinal epithelium from bacterial and other pathogens infection. We measured the amount of secretory IgA in the colon of DSS mice in both treated and untreated mice. Secretory IgA increased significantly after treatment with glucan extracts, indicating a higher protective status as is expected in the healthy intestine (Figure 5b). No significant differences were detected in animals treated with whole mill from stalk before DSS and after DSS without pretreatment.

## 3. Discussion

This study concentrated on evaluating the functional and spatial variation of glucan type and concentration in the edible mushrooms *P. eryngii*. In line with our previous publication [10], we demonstrated that the stalks (stipe) of the fruit body contained higher glucan content than the caps (pileus). Similarly, Park and associates [11] have recently demonstrated that the β-glucan contents were also higher in stipes of *P. eryngii*, claiming that this result is probably associated with the development of the major structural compounds of cell wall in mushrooms allowing for optimal supporting effect of the cap. Since β-glucans were repetitively demonstrated in a myriad of studies [2,12,13,14,15,16,17,18] to induce immunostimulatory events in mammalian cells, we hypothesized that this spatial variation may also impinge on the functionality of the different parts of *P. eryngii*.

In the present study, we measured α-glucan, β-glucan, and total glucan concentrations in mill preparations and compared these to extracted glucan lyophilized fractions from caps and stalks prepared from *P. eryngii* mushrooms. Overall, the net α-glucan, β-glucan, and total glucan concentrations were significantly higher in the extracted lyophilized glucan fraction from *P. eryngii* stalks (Table 1). Additionally, the α-glucan concentration was notably higher in extracted glucan fractions. Therefore, we hypothesized that glucan extracts from *P. eryngii* stalks should be significantly more effective than those from *P. eryngii* caps since, per gram of extract, there are more glucans (β-glucans and significantly more α-glucans).

We tested the effect of our glucan samples on LPS-induced production of TNF-α. The results demonstrated that glucan extracts from caps and stalk were more effective than mill mushroom preparations (Figure 1), and the effectiveness of stalks was slightly more pronounced. These results indicated that *P. eryngii* glucans prepared from either part of this edible mushroom could be used as an adequate treatment aimed to control diseases associated with TNF-α overproduction resulting from LPS-mediated inflammation.

In order to demonstrate the putative immunomodulatory effect of *P. eryngii* glucans derived from caps and stalks, we tested whether there are some differential abilities of these glucans to bind to glucan receptors associated with the inflammatory processes expressed on membrane of immune cells. To accomplish this aim, we tested the putative competitive of these glucans towards Dectin-1 and complement-3 (CR-3) receptors. Dectin-1 is a transmembrane protein widely expressed in the cell surface of neutrophils, macrophages, and dendritic cells, and shown to specifically bind β-glucans secreted by edible mushrooms [8]. We demonstrate herein that cap and stalk glucans from mill or isolated glucan dose competed similarly with anti-Dectin-1 antibody, indicating that both structural parts of *P. eryngii* competed for binding. Nonetheless, competition of glucans prepared from glucan extracts were more efficient than glucans from mill preparations (this was strikingly evident at higher concentrations of added glucans). Since the human β-glucan receptor is functionally equivalent to murine Dectin-1 [19] testing the *P. eryngii* derived glucans in human and mouse cell lines broadness the inter-species importance of these molecules.

Similar results were obtained CR-3 receptor. CR3 receptor is a heterodimeric transmembrane glycoprotein receptor consisting of a β2 subunit (CD18), and bound to a αM subunit (CD11b) [8]. The CR3 receptor is expressed on immune-associated cells such as macrophages or NK cells and on leukocytes. Similarly to Dectin-1, stalk and cap glucan preparations from *P. eryngii* did not significantly differ in their binding ability, indicating that glucans from both mushroom parts can compete with the glucan receptor CR3. These results may stress the importance of the interactions between the wide varieties of glucan structures with cells of the immune system. The binding may affect the immune system and keep it alert to fight pathogens or other opportunistic infections.

Next, we tested the effect of glucan preparations as an in vivo anti-inflammatory treatment for IBD. For IBD induction, we used the DSS mice model, which is an important animal research tool that has enormously contributed to our understanding of pathogenetic pathways associated with IBD [20]. Additionally, drugs used to treat human IBD also ameliorate symptoms of DSS-induced mouse IBD [21]. Histologic damage score includes colonic crypt alteration, epithelial cell injury, ulcer formation, and infiltration of inflammatory cells into the colonic lamina propria. Glucan extracts were equally efficient in diminishing histologic damage scores in the DSS-IBD model (Figure 3); however, mill preparations were ineffective. We assume that the concentrations or the specific mix of glucans (especially α-glucans; see Table 1) were more effective and contributed to their impressive ameliorating effect.

We measured the intestinal inflammatory response to the treatment of mill preparations of the DSS-IBD model compared to extracted glucan by assessing pro-inflammatory cytokine transcript levels in the intestinal tissue by RT-PCR. First, we clearly demonstrated that mill and glucan extracts were very effective in downregulating IFN-γ levels; however, glucan extracts were more effective than mill preparations (see Figure 4a). A striking effect was notable following treatment with stalk preparations compared to caps. This is a very interesting and important result if we take into consideration that the significant downregulation of a pro-inflammatory cytokine IFN-γ probably initiated the inflammation in the DSS-IBD model. Ito et al. [22] demonstrated that IFN-γ plays an essential role in the initiation of colitis induced by DSS treatment; additionally, several chemokines are produced in response to triggering in an IFN-γ-dependent manner.

These observations were followed up by measurements of MIP-2 intestinal transcript levels. MIP-2 is specifically expressed in the inflamed areas of the colon in patients with IBD [23] and plays an indispensable role in its development and progression. MIP-2 is produced by a wide variety of cells and plays a role as a powerful chemoattractant for immune cells such as monocytes, T and B cells, and others and thus intensifies the immune-mediated response in colitis. We assumed that MIP-2 is regulated in an IFN-γ-dependent manner and, as such, the effects on the cytokine levels by *P. eryngii* glucan treatment were similar to those exerted on IFN-γ levels. Indeed, mill and glucan extracts were very effective in downregulating MIP-2 levels; and as for IFN-γ, glucan extracts were more effective than mill preparations and stalk glucans were more effective than cap glucans (see Figure 4b).

We also measured the effect on CXCL1 levels. CXCL-1 levels are associated with recruitment of neutrophils into colonic tissues after that the inflammation process is initiated. The response of treatment to mill preparations compared to extracted glucan lyophilized fractions from caps and stalks prepared from *P. eryngii* mushrooms differed to the response of IFN-γ and MIP-2. Mill glucans were ineffective and glucans extracts from both caps and stalks were equally effective (see Figure 4c). We assumed that CXCL1 chemokines participate in later stages of inflammation, therefore controlling their levels was less essential than controlling the effector IFN-γ levels and its downstream response cytokine MIP-2.

CD14/CD16 monocytes represents a major proinflammatory immune cell population in IBD in general and in Crohn’s disease specifically [24]. Various researchers have demonstrated that peripheral CD14^+^/CD16^+^ cells play a key role as potential disease indicators and drug targets for anti-inflammatory treatment [25].

In the present study, we demonstrated that mill glucans were ineffective in regulating peripheral CD14^+^/CD16^+^ cell levels similarly to their effects on CXCL1 chemokines intestinal expression. On the other hand, glucan extracts from both caps and stalks were equally effective in regulating the number of CD14^+^/CD16^+^ monocytes, indicating that they induce a systemic anti-inflammatory effect (see Figure 5a). We assumed that CD14^+^/CD16^+^ cells, like CXCL1 chemokines, participated in later stages of inflammation.

One cause of IBD is an abnormally amplified immune response of the intestinal mucosal to the normal intestinal microbiome. IgA is the primary antibody present in intestinal secretions and its role is to stop unwanted pathogenic organisms reaching the intestinal epithelial cell surface. Additionally, IgA deficiency in humans has been closely associated with ulcerative colitis [25]. Our results indicated that intestinal disorders induced by DSS-treatment in mice also involved downregulation of IgA expression (see Figure 5b). Glucan extracts upregulated the levels of IgA to almost normal levels; however, mill preparations were found to be almost ineffective in this regard. We believe that either glucan administration prepared from caps or stalks of *P. eryngii* exerts an important effect in the preservation of the epithelial integrity and greatly contributes to the normal homeostasis phenotype of the intestinal epithelium.

## 4. Materials and Methods

### 4.1. Preparation of Whole Mill and Glucan Extractions from Caps and Stalks from P. eryngii

*P. eryngii* mushrooms were grown at the Matityahu Experimental Farm (Upper Galilei, Israel). Fresh mushrooms were harvested when they reached similar cap opening and color. After collection, *P. eryngii* were dissected into caps and stalks, the different parts were weighed, dried and milled to pass a 1.0 mm screen using a Retsch centrifugal mill preparations unit. This preparation provided the whole mill. Dried powder of these whole mill preparations were subsequently used for glucan extraction similarly to the methodology used in our previous publication [10]. Glucan extracts were lyophilized as we previously described [10] and dried pre-weighted glucan extract samples were used for further glucan analyses.

### 4.2. Glucans Analysis

Glucan concentrations of mill and glucan extracts from *P. eryngii* caps and stalks were determined using a mushroom and yeast-specific β-glucan kit (Megazyme International, Wicklow, Ireland) based on a colorimetric reaction using the previously described method [10]. The absorbance of the resulting color complex was measured using a spectrophotometer (Synergy 2, Multi-Mode Reader, BioTek, Winooski, VT, USA) at 510 nm. Total glucan (% *w*/*w* or g/100 g), α-glucan (% *w*/*w*) and β-glucan content (% *w*/*w*) were measured as we recently described [10].

### 4.3. TNF-α Secretion from LPS- and Glucan-treated Mouse Macrophage Cell Line J774.2

Mouse macrophage J774.2 cells (1 × 10^6^/well) were incubated with LPS (100 ng/mL) and different concentrations of glucans from mill or isolated glucans (dissolved in PBS under mild heating i.e., 60°C) for 24 h. Supernatants were collected at different time intervals (4, 6, 12, and 24 h) and evaluated for TNF-α secretion by ELISA employing anti-mouse TNF-α kit (R&D Systems, Minneapolis, MN, USA) and expressed as pg/mL. Glucan treatment did not affect cell viability as determined by MTT (3-(4, 5-dimethylthiazolyl-2)-2,5-diphenyltetrazolium bromide) assay as previously described [5].

### 4.4. Inhibition of CR3 and Dectin-1 Staining by Mill and Glucan Extracts from P. eryngii Caps and Stalks

We evaluated the ability of mill and glucan extracts to compete with specific FITC-labeled antibodies to CR3 (MN-41) or Dectin-1 (CLEC7A) using flow cytometry. To this end, we incubated human neutrophils (in case of CR3) and RAW 264.7 cell lines (in case of Dectin-1) with glucans (0.00025%; 0.0025%; 0.025%; i.e., 0.25 or 2.5 or 25 µg/1 mL media) of each preparation for 45 min at 4 °C then, after washing, we incubated the cells with each of the FITC-labeled antibodies for 30 min on ice. Flow cytometry was performed with a FACScan (Becton Dickinson, San Jose, CA, USA) flow cytometer and the data from over 10,000 cells/sample were analyzed. Results are specified as percentage of inhibition of staining.

### 4.5. Animals

Female, 8-week-old BALB/c mice (5–10 mice/group) were purchased from the Jackson Laboratory (Bar Harbor, ME, USA). The protocol for the research project was approved by the University of Louisville IACUC Committee and conformed to the provisions of the Declaration of Helsinki (as revised in Edinburgh 2000). Animals were sacrificed by CO_2_ asphyxiation followed by cervical dislocation.

### 4.6. Acute Colitis Induction in Mice

Dextran sodium sulfate (DSS) 3% (*w*/*v*) in water was provided in the drinking water for 7 days essentially as previously described [6]. Glucans were used simultaneously with the start of the treatment at dose 1 mg glucan/kg mice BW dissolved in PBS similarly to in vitro assay. The glucan treatment continued until day 16. Glucans treatment was provided by gavage. Disease severity was evaluated at the end of the study based on evaluation of histology of the colon (histologic damage score). Histology damage score was performed essentially as we previously described [6]. After killing, the distal third of the colon was transferred to a 4% buffered formalin solution. Histological scoring of the fixed (paraffin-embedded), sectioned and haematoxylin and eosin-stained tissues was performed in a blinded fashion essentially as previously described by Dieleman et al. [26]. Scoring was performed according to the following criteria: inflammation (0 (none) to 4 (severe)), extent (0 (none) to 4 (transmural)) and crypt damage (0 (none) to 4 (entire crypt and epithelium lost)). The three scores were summed to give a total score (0–12). Grading was performed by two investigators blinded to the treatment groups.

### 4.7. Monocytes Staining

Mice blood samples were obtained from control, DSS-treated, and glucan and DSS-treated mice. Inflammatory monocytes (CD14^+^/CD16^+^) were stained using anti-CD14-FITC and anti-CD16-PE antibodies. 20 µL of whole blood was incubated with the labeled antibodies and washed. Following application of 300 µL erythrocyte lysing buffer, the cells were washed and analyzed by flow cytometry.

### 4.8. RNA Preparation and Real-time PCR

Total RNA was isolated from colonic tissue (snap frozen in liquid N_2_) using the RNeasy Plus Mini Kit (Qiagen, Santa Clarita, CA, USA) as described in the manufacturer’s protocol. RNA concentration was quantified by ultraviolet spectrophotometry at 260 nm, and the purity and integrity were determined using a NanoDrop (Thermo Fisher Scientific, Wilmington, DE, USA).

RT real-time PCR assays were performed to quantify steady-state mRNA levels of selected molecules CXCL1, IFN-gamma, and MIP-2. cDNA was synthesized from 0.2 μg of total RNA. Real-time PCR amplification was performed using Primer Express Software (Applied Biosystems, Foster City, CA, USA). Target probe was labeled with fluorescent reporter dye. PreDeveloped TaqMan primers and probes were used for the detection. Reporter dye emission was detected by an automated sequence detector combined with ABI Prism 7700 Sequence Detection System software (Applied Biosystems). Real-time PCR quantification was then performed using TaqMan 18S rRNA controls as described [27]. Gene expression was expressed as fold increase relative to negative control.

The primers used for real-time PCR were as shown in Table 2:

### 4.9. Secretory Intestinal IgA

Elisa plates were coated with anti-IgA antibodies, incubated with fecal samples from all treatment mice groups, and diluted 1:100 in PBS. IgA was developed using anti-IgA-biotin and HRP-streptavidin as previously reported [28].

### 4.10. Statistical Analyses

Statistical analyses were performed by one-way repeated-measure ANOVA with Tukey–Kramer or Dunnett’s test. Results are presented as mean ± SD. All figures show representative results of at least two independent experiments.

## 5. Conclusions

We demonstrate that the most effective glucans in ameliorating IBD-associated symptoms induced by DSS treatment in mice were glucan extracts prepared from the stalk of *P. eryngii*. We believe that these spatial distinctions may be helpful in selecting more effectively appropriate medicinal mushrooms.

## Figures and Tables

**Table 1:** Glucan content in cap and stalks from *P. eryngii* mill and glucan extracts. α, β, and total glucan concentrations (g/100 g dried matter) in mills and glucan extracts prepared from cap and stalks harvested from *P. eryngii* mushrooms. * *p* < 0.001, ** *p* < 0.0001 comparison between caps to respective stalks.

*P. eryngii* Parts and Preparations	α-Glucan (g/100 g)	β-Glucan (g/100 g)	Total Glucans (g/100 g)
Cap whole mill	0.804 ± 0.006	29.519 ± 0.98	30.32 ± 0.5
Stalk whole mill	4.505 ± 0.35 *	38.412 ± 1.2 *	42.92 ± 0.77 *
Cap glucan extract	6.545 ± 1.38	21.804 ± 1.27	28.5 ± 1.32
Stalk glucan extract	16.9851 ± 1.6 **	29.807 ± 2.6 *	46.79 ± 2.12 *

**Table 2:** Primer sequences used for RT-PCR.

Gene	Forward Primer	Reverse Primer
*MIP-2*	5′-TGGGTGGGATGTAGCTAGTTCC	5′-AGTTTGCCTTGACCCTGAAGCC
*Cxcl1*	5’- GCCACACTCAAGAATGGTCG	5’-TGGGGACACCCTTTAGCATC
*INF-γ*	5’-GTCTCTTCTTGGATATCTGGAGGAACT	5’-GTAGTAATCAGGTGTGATTCAATGACGC

## References

[1] Ma G., Kimatu B.M., Zhao L., Yang W., Pei F., Hu Q. (2018). Impacts of Dietary *Pleurotus eryngii* Polysaccharide on Nutrient Digestion, Metabolism, and Immune Response of the Small Intestine and Colon-An iTRAQ-Based Proteomic Analysis. Proteomics.

[2] Kim Y.H., Jung E.G., Han K.I., Patnaik B.B., Kwon H.J., Lee H.S., Kim W.J., Han M.D. (2017). Immunomodulatory Effects of Extracellular beta-Glucan Isolated from the King Oyster Mushroom *Pleurotus eryngii* (Agaricomycetes) and Its Sulfated Form on Signaling Molecules Involved in Innate Immunity. Int. J. Med. Mushrooms.

[3] Xu D., Wang H., Zheng W., Gao Y., Wang M., Zhang Y., Gao Q. (2016). Charaterization and immunomodulatory activities of polysaccharide isolated from *Pleurotus eryngii*.. Int. J. Biol. Macromol..

[4] Zhu F., Du B., Xu B. (2016). A critical review on production and industrial applications of β-glucans. Food Hydrocoll..

[5] Lavi I., Friesem D., Geresh S., Hadar Y., Schwartz B. (2006). An aqueous polysaccharide extract from the edible mushroom *Pleurotus ostreatus* induces anti-proliferative and pro-apoptotic effects on HT-29 colon cancer cells. Cancer Lett..

[6] Lavi I., Levinson D., Peri I., Nimri L., Hadar Y., Schwartz B. (2010). Orally administered glucans from the edible mushroom *Pleurotus pulmonarius* reduce acute inflammation in dextran sulfate sodium-induced experimental colitis. Br. J. Nutr..

[7] Lavi I., Nimri L., Levinson D., Peri I., Hadar Y., Schwartz B. (2012). Glucans from the edible mushroom *Pleurotus pulmonarius* inhibit colitis-associated colon carcinogenesis in mice. J. Gastroenterol..

[8] Legentil L., Paris F., Ballet C., Trouvelot S., Daire X., Vetvicka V., Ferrieres V. (2015). Molecular Interactions of β-(1-->3)-Glucans with Their Receptors. Molecules.

[9] Yamamoto-Furusho J.K. (2018). Inflammatory bowel disease therapy: Blockade of cytokines and cytokine signaling pathways. Curr. Opin. Gastroenterol..

[10] Avni S., Ezove N., Hanani H., Yadid I., Karpovsky M., Hayby H., Gover O., Hadar Y., Schwartz B., Danay O. (2017). Olive Mill Waste Enhances α-Glucan Content in the Edible Mushroom *Pleurotus eryngii*.. Int. J. Mol. Sci..

[11] Earnshaw S.R., McDade C.L., Chu Y., Fleige L.E., Sievenpiper J.L. (2017). Cost-effectiveness of Maintaining Daily Intake of Oat β-Glucan for Coronary Heart Disease Primary Prevention. Clin. Ther..

[12] Chan G.C., Chan W.K., Sze D.M. (2009). The effects of β-glucan on human immune and cancer cells. J. Hematol. Oncol..

[13] Guerra Dore C.M., Azevedo T.C., De Souza M.C., Rego L.A., De Dantas J.C., Silva F.R., Rocha H.A., Baseia I.G., Leite E.L. (2007). Antiinflammatory, antioxidant and cytotoxic actions of beta-glucan-rich extract from Geastrum saccatum mushroom. Int. Immunopharmacol..

[14] Nosal’ova V., Bobek P., Cerna S., Galbavy S., Stvrtina S. (2001). Effects of pleuran (β-glucan isolated from *Pleurotus ostreatus*) on experimental colitis in rats. Physiol. Res..

[15] Soltanian S., Stuyven E., Cox E., Sorgeloos P., Bossier P. (2009). Beta-glucans as immunostimulant in vertebrates and invertebrates. Crit. Rev. Microbiol..

[16] Vetvicka V., Vetvickova J. (2018). Glucans and Cancer: Comparison of Commercially Available β-glucans—Part IV. Anticancer Res..

[17] Volman J.J., Helsper J.P., Wei S., Baars J.J., Van Griensven L.J., Sonnenberg A.S., Mensink R.P., Plat J. (2010). Effects of mushroom-derived β-glucan-rich polysaccharide extracts on nitric oxide production by bone marrow-derived macrophages and nuclear factor-κB transactivation in Caco-2 reporter cells: Can effects be explained by structure?. Mol. Nutr. Food Res..

[18] Wang Q., Sheng X., Shi A., Hu H., Yang Y., Liu L., Fei L., Liu H. (2017). β-Glucans: Relationships between Modification, Conformation and Functional Activities. Molecules.

[19] Willment J.A., Marshall A.S., Reid D.M., Williams D.L., Wong S.Y., Gordon S., Brown G.D. (2005). The human β-glucan receptor is widely expressed and functionally equivalent to murine Dectin-1 on primary cells. Eur. J. Immunol..

[20] Eichele D.D., Kharbanda K.K. (2017). Dextran sodium sulfate colitis murine model: An indispensable tool for advancing our understanding of inflammatory bowel diseases pathogenesis. World J. Gastroenterol..

[21] Wirtz S., Neurath M.F. (2007). Mouse models of inflammatory bowel disease. Adv. Drug Deliv. Rev..

[22] Ito R., Shin-Ya M., Kishida T., Urano A., Takada R., Sakagami J., Imanishi J., Kita M., Ueda Y., Iwakura Y. (2006). Interferon-γ is causatively involved in experimental inflammatory bowel disease in mice. Clin. Exp. Immunol..

[23] Neurath M.F. (2014). Cytokines in inflammatory bowel disease. Nat. Rev. Immunol..

[24] Hanai H., Iida T., Takeuchi K., Watanabe F., Yamada M., Kikuyama M., Maruyama Y., Iwaoka Y., Hirayama K., Nagata S. (2008). Adsorptive depletion of elevated proinflammatory CD14+CD16+DR++ monocytes in patients with inflammatory bowel disease. Am. J. Gastroenterol..

[25] Koch S., Kucharzik T., Heidemann J., Nusrat A., Luegering A. (2010). Investigating the role of proinflammatory CD16+ monocytes in the pathogenesis of inflammatory bowel disease. Clin. Exp. Immunol..

[26] Dieleman L.A., Palmen M.J., Akol H., Bloemena E., Pena A.S., Meuwissen S.G., Van Rees E.P. (1998). Chronic experimental colitis induced by dextran sulphate sodium (DSS) is characterized by Th1 and Th2 cytokines. Clin. Exp. Immunol..

[27] Vetvicka V., Volny T., Saraswat-Ohri S., Vashishta A., Vancikova Z., Vetvickova J. (2007). Glucan and resveratrol complex—Possible synergistic effects on immune system. Biomed. Pap. Med. Fac. Univ. Palacky Olomouc Czech..

[28] Cao A.T., Yao S., Gong B., Elson C.O., Cong Y. (2012). Th17 cells upregulate polymeric Ig receptor and intestinal IgA and contribute to intestinal homeostasis. J. Immunol..

